# Evaluation of oxidative stress in acute pancreatitis

**DOI:** 10.3389/fimmu.2026.1736996

**Published:** 2026-05-15

**Authors:** Vlad Pădureanu, Dan Nicolae Florescu, Sergiu Marian Cazacu, Virginia Maria Rădulescu, Rodica Pădureanu, Mihaela Andreea Podeanu, Radu Razvan Mititelu, Mihnea Marian Pomacu, Ana Maria Bugă

**Affiliations:** 1 Department of Internal Medicine, University of Medicine and Pharmacy of Craiova, Craiova, Romania; 2 Department of Gastroenterology, University of Medicine and Pharmacy of Craiova, Craiova, Romania; 3 Department of Medical Informatics and Biostatistics, University of Medicine and Pharmacy of Craiova, Craiova, Romania; 4 Department of Pediatrics, University of Medicine and Pharmacy of Craiova, Craiova, Romania; 5 Department of Microbiology, University of Medicine and Pharmacy of Craiova, Craiova, Romania; 6 Department of Biochemistry, University of Medicine and Pharmacy of Craiova, Craiova, Romania

**Keywords:** acute pancreatitis, aggregate index of systemic inflammation (AISI), caspase-3, C-reactive protein (CRP), glutathione (GSH), oxidative stress, protein carbonyls, redox imbalance index (RII)

## Abstract

**Background:**

Oxidative stress is implicated in acute pancreatitis (AP), but its diagnos-tic/prognostic value at presentation is uncertain; this study evaluated whether redox markers discriminate radiologic severity and how they relate to inflammation and apoptosis.

**Methods:**

In this observational study cohort severity was classified as Balthazar D vs A–C score. Assays biomarkers included protein carbonyls (P-carb), lipid peroxidation state (TBARS), antioxidant status (TAC), reduced glutathione (GSH), catalase (CAT); inflammatory markers (CRP, AISI, SIRI); pro-apoptosis/innate immunity markers (caspase-3, IL-1β, AIM2). Discrimination used ROC/AUC with bootstrap CIs; a prespecified Redox Imbalance Index (RII) and exploratory load-vs-buffer ratios were derived. Correlations employed Spearman and age-adjusted partial Spearman with Benjamini Hochberg–FDR correction; logistic models used z-scored predictors with.632 bootstrap correction.

**Results:**

Single-marker discrimination was modest (GSH AUC of 0.57 [95% CI 0.24–0.85]; CRP of 0.55 [0.26–0.82]; AISI of 0.53 [0.30–0.75]); RII of 0.42 [0.17–0.69]; derived indices (POBI ≤ 0.48). Combined models overfit (apparent AUC 0.56–0.59;.632 corrected ≈0.39). By contrast, caspase-3 correlated with CRP, GSH, and P-carb (moderate, age-robust), and GSH–P-carb coupling was strong, whereas TBARS aligned weakly.

**Conclusions:**

At presentation, oxidative-stress signatures co-vary with inflammation and apoptosis (strong GSH–P-carb coupling and moderate, age-robust caspase-3 correlations with CRP and P-carb). However, neither single markers nor composite indices (including the prespecified Redox Imbalance Index) reliably discriminated radiologic severity (AUCs 0.42–0.57, all 95% CIs including 0.50). Multivariable models showed substantial overfitting after.632 bootstrap correction. These findings support further investigation of the antioxidant–protein-oxidation and apoptosis–inflammation axes in adequately powered, longitudinal studies rather than as immediate clinical triage tools.

## 1 Introduction

Acute pancreatitis (AP) is one of the most encountered gastrointestinal diseases resulting in hospitalization (1, 2). It is a condition defined by the acute inflammation of the pancreas (3) and, according to the Atlanta classification, severity is classified as mild, moderate or severe (4).

Most commonly AP is determined by bile stones or excessive alcohol consumption (5). There are many other less common causes of acute pancreatitis including toxins, drugs, infections, trauma, vascular insults and anatomic abnormalities (6), but the third cause incriminated in this pathology, even if the involved mechanism cannot be fully explained, is hypertriglyceridemia (7). The most prevalent type of hypertriglyceridemia is mild hypertriglyceridemia, which is linked to visceral obesity, metabolic syndrome, and diabetes (8).

The World Health Organization (WHO) estimates that over 1 billion people worldwide—650 million adults, 340 million adolescents, and 39 million children— suffer from obesity (9). Metabolic syndrome (MetS) prevalence has increased drastically since the obesity epidemic (10) and it is an important driver of the current global cardiovascular crisis (11). It dramatically raises the risk of cardiovascular disease (CVD), type 2 diabetes, and sudden death. Obesity, insulin resistance, high blood pressure, decreased levels of serum high density cholesterol (HDL-C), and elevated levels of serum triglycerides are among the metabolic dysregulations that constitute metabolic syndrome (12). There are studies that proofed the existence of a low-grade inflammation and proinflammatory state associated with MetS (13, 14).

During an episode of AP, acinar cells undergo a range of changes, starting with the early activation of pancreatic proteolytic enzymes, which results in cell damage and gland self-digestion (15). By releasing inflammatory cytokines and chemokines facilitating the recruitment and activation of circulating neutrophils and macrophages, injured acinar cells start an inflammatory response. High concentrations of oxidants and cytotoxic agents are released by activated neutrophils, exacerbating the pancreatic damage. As neutrophils transmigrate through endothelial cells, local inflammation develops into a systemic inflammatory response syndrome (SIRS) (13, 16). Pro-inflammatory cytokines and chemokines are important mediators of the innate immune system. Interleukins (IL) 1β, 6, 8, 18 and tumor necrosis factor alpha (TNFα) are reported by some studies to be markers of severity in AP (17). Cellular IL1β and IL18 need to be cleaved to be activated into their biologically active forms (18). This job is done by an assembly of a multi-protein signaling platform called the inflammasome (13, 19), which consist of an upstream sensor protein (belonging to the NLR or the ALR family), and an adaptor protein—the apoptosis-associated speck-like protein containing a caspase recruitment domain (ASC) (20). These are a mixture of intracellular protein complexes that facilitate the autocatalytic activation of inflammatory caspases, which trigger proinflammatory cell death mechanisms such as pyroptosis and the release of cytokines and alarmins into the bloodstream, hence driving host and immunological responses (21). The NLR pyrin domain-containing protein 3 (NLRP3) inflammasome, has emerged as the most versatile and well-characterized inflammasome, consisting of an intracellular multi-protein complex that acts as a central driver of inflammation (22). It is considered a general sensor of cellular damage that responds to both Pathogen-Associated Molecular Patterns (PAMPs), and endogenous molecules released from damaged cells, called Damage-Associated Molecular Patterns (DAMPs) (23). As in AP pathogenesis there is a sterile inflammation, the capacity of the NLRP3 to recognize DAMPs confers it a primordial importance (13, 15).

We aimed to characterize oxidative (P-carb, TBARS), antioxidant (TAC, CAT, GSH), apoptotic (caspase-3), and inflammasome-related (IL-1β, Absent in melanoma 2 -AIM2) markers at presentation in acute pancreatitis, to test their association with radiologic severity (Balthazar), and to evaluate discrimination (Receiver Operating Characteristic Curve - ROC) including composite indices (RII) and combined models. Exploratory analyses assessed gender- and age-related heterogeneity and metabolic phenotype (pancreatitis vs pancreatitis+MetS). We hypothesized small-to-moderate effect sizes and limited single-marker discrimination.

## 2 Materials and methods

### 2.1 Study design and population

This study is a single centre observational pilot study designed to explore the correlation of oxidative stress and pro inflammatory markers with diseases severity in pancreatitis. The study cohort included consecutive adults presenting with pancreatitis to Emergency Hospital of Craiova, Romania. Radiologic severity was graded on cross-sectional imaging using the Balthazar classification (A–E). The primary analysis contrasted severe (D) versus non-severe (A–C) at presentation. Because of class imbalance and occasional quasi-separation in multivariable settings, an alternative split (C/D vs B) was specified *a priori* for interaction models and “Oxidative Burden” analyses. Acute-on-chronic presentations were eligible and analyzed within the same framework; chronicity-stratified analyses were not performed due to small numbers. Etiology and key exposure variables (alcohol use, smoking, relevant medications) were abstracted from medical records when consistently documented; variables with incomplete capture were treated as limitations and used only in sensitivity analyses.

Ethics. The study complied with the Declaration of Helsinki and was approved by the Ethics Committee of the University of Medicine and Pharmacy of Craiova (160/19 August 2022). Patient consent procedures followed institutional policy.

### 2.2 Clinical and laboratory data

Demographic data (age, sex) and clinical variables at presentation were abstract-ed from the medical record, including radiologic grade and comorbidity history (e.g., hypertension). A metabolic phenotype was recorded as pancreatitis only versus pancreatitis + MetS (MetS per institutional/consensus criteria). Metabolic syndrome was defined using [IDF/NCEP-ATP III/institutional criteria—specify explicitly], requiring [list the components briefly]. Given the small MetS stratum, analyses by metabolic phenotype were considered exploratory.

Age groups. Age was dichotomized *a priori* as ≤55 vs >55 years for interaction/sensitivity analyses, reflecting higher clinical risk ≥55 and yielding balanced strata in this cohort. Additional potential confounders were extracted where available: suspected etiology (biliary, alcohol-related, hypertriglyceridemia, idiopathic/other), smoking status (current/former/never or yes/no), alcohol consumption (clinically significant yes/no or categorical), and pre-admission medication classes relevant to redox/inflammation (e.g., statins, antidiabetic drugs, antioxidant supplements, corticosteroids/immunosuppressants). Comorbidity burden was summarized both as individual conditions (e.g., diabetes, hypertension) and as a simple count (0/1/≥2) for parsimonious adjustment.

### 2.3 Measurement of biomarkers

Venous blood was drawn at admission, prior to specific therapy when feasible. Routine chemistry and hematology followed institutional standard operating procedures. The dataset included CRP (mg/L) and complete blood count parameters used to compute composite inflammatory indices. Units follow laboratory reports and are preserved throughout tables and analyses.

All blood sample analyses were conducted in a single laboratory using standardized protocols and commercially available assay kits according to the manufacturers’ instructions, which minimizes interlaboratory variability. Samples were processed using uniform preanalytical procedures, and all measurements were conducted by trained personnel. Serum/plasma were separated by centrifugation and stored at −80 °C until further analysis.

Venous blood was collected at emergency department admission, as close as possible to patient presentation and prior to specific therapeutic interventions. Time from symptom onset to blood sampling was not systematically recorded in all cases and is therefore noted as a limitation.

#### 2.3.1 Oxidative stress biomarkers

Protein carbonyls (P-carb) assay was performed by standard spectrophotometric method using 2,4-dinitrophenylhydrazine (DNPH) (24, 25). The PCARB content was determined according with the molar extinction coefficient of DNFH (22,000 M−1 cm−1). P-carb were quantified and expressed in μmol/L and normalized to μmol/g protein where applicable; TBARS were measured by standard spectrophotometric TBARS (MDA) method (23–25). The TBARS concentration in human deproteinized plasma was calculated according with using the molar extinction coefficient of MDA (1.55 × 105 M−1 cm−1) and expressed as index of lipid peroxidation (μmol/mL). TAC assay was performed by spectrophotometry using 2,2 diphenyl-1-picrylhydrazyl (DPPH) method (25, 26). TAC was expressed in μmol DPPH/L; GSH was measured from RBC lysate by 5′-dithio-bis-(2-nitrobenzoic acid) (DTNB) spectrophotometric method (24, 25). GSH concentration was calculated using using the GSH standard curve and ex-pressed as μmol/L; CAT activity was determined by Aebi method, a standard spectro-photometric method (27–29). The CAT ac-tivity was expressed in U/mL.

#### 2.3.2 Apoptosis and inflammation biomarkers

Caspase-3 assay was performed by Human Caspase 3 ELISA kit, Biorbyt (orb385276) according to manufacturer instructions. IL-1β assay was performed usingHuman IL-1β ELISA kit, Biorbyt (orb735138) according to manufacturer instructions. AIM2 level were assayed by Human Interferon-Inducible Protein AIM2 ELISA kit, MyBioSource (MBS9714300) according to manufacturer instructions.

### 2.4 Outcomes

The primary outcome was radiologic severity at presentation (D vs A–C). Sec-ondary/ancillary analyses examined the alternative C/D vs B split, prespecified age in-teractions (Age×GSH, Age×TAC), and a data-driven oxidative score from principal component analysis (PCA).

### 2.5 Data handling and pre-processing

Continuous variables are reported as median [IQR] and categorical variables as n (%). Analyses were performed on complete-case sets per test/model; the effective sample size for each analysis is indicated in Results/Tables. Units and scaling follow laboratory outputs. Predictors entering multivariable models were z-scored.

### 2.6 Statistical analysis

Data handling and analyses were performed in IBM SPSS Statistics v26. Continuous variables are reported as median [IQR] and categorical variables as n (%). All tests were two-sided with α = 0.05. Analyses followed a complete-case approach per test/model; effective sample sizes are provided in the Results/Tables.

Group comparisons. Differences between D vs A–C were assessed using Mann–Whitney U tests for continuous variables. Where indicated, non-parametric effect sizes (e.g., Cliff’s delta) accompanied p-values. Categorical summaries used counts/percentages; Fisher’s exact tests were noted where informative but interpreted cautiously due to sparse cells.

Discrimination (ROC). Receiver operating characteristic (ROC) analyses reported AUC with bootstrap percentile 95% confidence intervals (4,000 resamples, SPSS Boot-strap). When relevant, the Youden J cut-off, sensitivity, specificity, and likelihood ratios (LR±) were derived. ROC analyses covered single markers (e.g., GSH, CRP, AISI, SIRI, P-carb, TAC, TBARS, caspase-3), the prespecified Redox Imbalance Index (RII), and exploratory redox ratios (POBI = z[P-carb_g] − z[GSH], LOBI = z[TBARS] − z[GSH]). An inflammation-independent oxidative-damage metric was evaluated via the rank residual of P-carb_g after regression on CRP (residual P-carb_g | CRP) fol-lowed by ROC.

Correlations. Associations among biomarkers were quantified with Spearman’s ρ; uncertainty was summarized with bootstrap percentile 95% CIs (4,000 resamples). Kendall’s τ was used in sensitivity checks. Age-adjusted partial Spearman was implemented by rank residualization (ranks of each variable residualized on age; Pearson correlation on residuals). To control multiplicity within prespecified correlation families (e.g., CASP3 with CRP/GSH/P-carb), the Benjamini–Hochberg false discovery rate (BH–FDR) was applied; q-values are reported alongside p-values.

Confounding control followed a parsimonious strategy due to the small sample size. Primary association analyses used age-adjusted partial Spearman correlations. Sensitivity analyses additionally adjusted for etiology and comorbidity burden (0/1/≥2) when available, using rank-residualization (partial correlations) and stratified checks. For predictive models, we limited multivariable logistic regression to small specifications (age, sex ± one clinical confounder, plus ≤2 biomarkers) to mitigate overfitting; optimism-corrected performance was assessed via.632 bootstrap. Variables not systematically captured in the medical record (e.g., smoking, alcohol, medication history) could not be fully controlled and are discussed as sources of residual confounding.

Multivariable modeling and validation. Logistic regression models for severity (D vs A–C) used z-scored predictors. Apparent AUCs and.632 bootstrap–corrected AUCs (SPSS Bootstrap with custom syntax) were reported to address optimism. Interaction terms were prespecified (Age×GSH, Age×TAC) and estimated on the C/D vs B split to mitigate class imbalance and quasi-separation; cross-validated performance for inter-action models is reported where available.

Principal component analysis (PCA). A PCA restricted to oxidative markers yielded an oxidative score (PC1), which was entered in age-adjusted logistic models. For this specification, k-fold cross-validation (k = 5) provided mean ± SD AUC on out-of-fold predictions.

Severity splits. The primary contrast was D vs A–C. Where explicitly stated (in-teraction-oriented and Oxidative Burden analyses), the C/D vs B split was used to im-prove numerical stability under sparse/separated outcomes.

*Post-hoc* power estimation. Given the observed small-to-modest effect sizes (Cliff’s Δ ≤ 0.27 for most markers) and the variability in the present cohort, approximately 80–120 total patients would be required to detect group differences in key markers (GSH, CRP) with 80% power at α = 0.05 using a two-sided Mann–Whitney U test. Detecting an AUC of 0.70–0.75 versus 0.50 would require substantially larger samples (typically 100–200 patients depending on prevalence of severe cases). These calculations confirm the pilot nature of the current study and the need for larger validation cohorts.

## 3 Results

### 3.1 Baseline characteristics and radiologic severity at presentation

This study analyzed a cohort of 25 patients with pancreatitis with or without associated MetS at presentation. Categorical baseline characteristics are summarized in **Table 1**: 18 (72.0%) were male and 7 (28.0%) were female. Primary diagnosis was acute pancreatitis in 21 (84.0%) and acute-on-chronic pancreatitis in 4 (16.0%). By BMI category, 9 (36.0%) were normal weight, 9 (36.0%) overweight, and 7 (28.0%) had grade-1 obesity. Based on metabolic phenotype, 16 (64.0%) were pancreatitis only and 9 (36.0%) were pancreatitis + MetS. A history of arterial hypertension was recorded in 14 (56.0%), while diabetes mellitus was present in 9 (36.0%), including type-2 diabetes in 4 (16.0%) (**Table 1**).

**Table 1:** Baseline characteristics—categorical variables and primary diagnosis (n=25).

Variable	Level	Value
		N (%)
Primary diagnosis	Acute pancreatitis	21 (84.00%)
	Acute–on–chronic pancreatitis	4 (16.00%)
Group	pancreatitis	16 (64.00%)
	pancreatitis + MetS	9 (36.00%)
Hypertension history	Yes	14 (56.00%)
	No	11 (44.00%)
MetS criteria	Yes	9 (36.00%)
	No	16 (64.00%)
BMI categories	normoweight	9 (36.00%)
	overweight	9 (36.00%)
	obesity grade 1	7 (28.00%)

The mean age was 54.84 ± 12.38 years (95% CI 49.73–59.95). The age distribution approximated normality (Shapiro–Wilk W = 0.930, p=0.088); accordingly, age is summarized as mean ± SD in the text, while continuous biomarkers are presented as medians in **Table 2** for comparability with non-normal variables.

**Table 2:** Baseline characteristics—continuous variables (n=25).

Variable	Median [IQR]
CRP (mg/L)	72.0 [26.9–98.0]
SIRI	5.10 [2.76–6.10]
NLR	5.67 [3.05–9.11]
AISI	1179.8 [467.0–1826.5]
TAG (mg/dL)	106 [75–214]
HDL-C (mg/dL)	44.7 [35.6–51.7]
Age (years)	54 [48–66]
ESR (mm/h)	25 [14–38]
Balthazar inflammation (grade points)	2.00 [1.00–3.00]

In the overall cohort, median [IQR] values were: CRP - 72.00 mg/L [26.90–98.00], SIRI 5.10 [2.76–6.10], neutrophile lymphocyte ratio (NLR) - 5.67 [3.05–9.11], AISI - 1179.80 [467.00–1826.50], triglycerides (TAG) - 106.00 mg/dL [75.00–214.00], high density lipoprotein -cholesterol (HDL-C) - 44.70 mg/dL [35.60–51.70], erythrocyte sedimentation ratio ESR - 25.00 mm/h [14.00–38.00], and Balthazar inflammation points 2.00 [1.00–3.00] (**Table 2**). Secondary diagnoses were heterogeneous but sparse; the most frequent labels were “Balthazar C” (n=4, 19.0%) and “Balthazar D” (n=4, 19.0%), followed by “previous history” (n=4, 19.0%) and “acute-on-chronic” (n=3, 14.3%), while other entries were ≤10%.

Balthazar inflammation points discriminated severe (D) vs non-severe (A–C) on the ROC scale (C-index 0.88, 95% CI 0.72–0.99), consistent with the distribution of values summarized in **Table 2**.

### 3.2 Severity comparisons and stratified analyses

Radiologic severity was defined as non-severe (A–C) versus severe (D), with 18 (72.0%) and 7 (28.0%) patients, respectively. Across demographic, inflammatory, and metabolic domains, between-group differences were small and non-significant. For in-stance, CRP was 68.98 mg/L [26.90–88.28] in non-severe vs 72.00 mg/L [40.44–121.47] in severe (p=0.745), while composite indices displayed comparable distributions (SIRI 5.20 [2.21–6.03] vs 4.36 [3.80–5.70], p=0.929; NLR 5.55 [3.01–10.14] vs 6.22 [3.65–7.88], p=0.836; AISI 1317.26 [383.21–1995.40] vs 1179.77 [809.46–1492.36], p=0.836). Meta-bolic parameters were also similar (triglycerides 99.00 [75.00–195.00] vs 131.00 [91.00–229.00] mg/dL, p=0.607; HDL-C 45.20 [37.80–50.50] vs 39.80 [35.50–54.60] mg/dL, p=0.790), and ESR tended to be lower in severe cases (32.50 [15.00–40.00] vs 19.00 [13.00–24.00] mm/h, p=0.348). Comparisons of clinical and inflammatory variables are summarized in **Table 3**, while oxidative, antioxidant, apoptotic, and inflammasome markers are presented in **Table 4**.

**Table 3:** Continuous clinical and inflammatory variables by radiologic severity (A–C vs D): median [IQR] and p (Mann–Whitney U).

Variable	Non-severe, median [IQR]	Severe, median [IQR]	P
Balthazar inflammation points	2.00 [1.00–2.00]	3.00 [3.00–3.00]	<0.001
CRP (mg/L)	68.98 [27.90–88.30]	72.00 [40.40–121.50]	0.745
SIRI (a.u.)	5.20 [2.21–6.03]	4.36 [3.80–5.70]	0.929
NLR	5.55 [3.01–10.14]	6.22 [3.65–7.88]	0.836
AISI (a.u.)	1317.30 [383.20–1995.40]	1179.80 [809.50–1492.40]	0.836
TAG (mg/dL)	99.00 [75.00–195.00]	131.00 [91.00–229.00]	0.607
HDL-C (mg/dL)	45.20 [37.80–50.50]	39.80 [35.50–54.60]	0.790
ESR (mm/h)	32.50 [15.00–40.00]	19.00 [13.00–24.00]	0.348
Age (years)	56.00 [46.00–66.00]	54.00 [51.00–60.00]	0.761

Values are median [IQR]; p from Mann–Whitney U. Age is additionally summarized as mean ± SD in text owing to approximate normality (Shapiro–Wilk p=0.088).

**Table 4:** Oxidative, antioxidant, apoptotic and inflammasome markers by radiologic severity (A–C vs D): median [IQR] and p (Mann–Whitney U).

Marker	Non-severe (A–C), median [IQR]	Severe (D), median [IQR]	P
P-carb (μmol/L)	5.91 [5.04–10.17]	5.45 [5.25–14.54]	0.976
P-carb (μmol/g protein)	91.47 [68.68–140.78]	75.83 [64.88–162.12]	0.976
TBARS (μmol/mL)	0.68 [0.59–1.04]	0.64 [0.59–0.73]	0.560
TAC (μmol DPPH/L)	54.91 [49.15–69.09]	57.41 [52.77–58.89]	0.930
CAT activity (U/mL)	4.69 [3.98–5.09]	4.97 [4.36–5.10]	0.570
GSH (μmol/L)	1.30 [1.05–1.50]	1.46 [1.01–1.66]	0.630
Caspase-3 (ng/mL)	7.37 [5.45–7.72]	7.07 [5.45–7.73]	1.000
IL-1β (pg/mL)	10.11 [9.37–11.60]	12.82 [8.62–19.91]	0.560
AIM2 (pg/mL)	857.15 [682.67–929.44]	772.86 [631.85–798.70]	0.320

Values are median [IQR]; p from Mann–Whitney U.

Extending the analysis to the oxidative, antioxidant, apoptotic, and inflammasome panel, between-group shifts remained modest and non-significant across analytes (all p>0.05). Medians [IQR] for P-carb, TBARS, TAC, CAT activity, GSH, caspa-se-3, IL-1β, and AIM2 are reported in **Table 4** (e.g., TBARS 0.68 [0.59–1.04] vs 0.64 [0.59–0.73] μmol/mL, p=0.560; TAC 54.91 [49.15–69.09] vs 57.41 [52.77–58.89] μmol DPPH/L, p=0.930; GSH 1.30 [1.05–1.50] vs 1.46 [1.01–1.66] μmol/L, p=0.630; IL-1β 10.11 [9.37–11.60] vs 12.82 [8.62–19.91] pg/mL, p=0.560; AIM2 857.15 [682.67–929.44] vs 772.86 [631.85–798.70] pg/mL, p=0.320).

Correlation between apoptosis and redox/inflammation markers. Prespecified analyses showed that caspase-3 correlated positively with CRP (Spearman ρ=0.542, p=0.005; BH–FDR q=0.010), GSH (ρ=0.546, p=0.005; q=0.010), and protein carbonyls expressed both per liter (ρ=0.443, p=0.027; q=0.027) and per gram of protein (ρ=0.457, p=0.022; q=0.027). These associations remained significant after age-adjusted partial Spearman on ranks (ρ_partial=0.49–0.50; p=0.010–0.013; q=0.017–0.024). Taken together, apoptosis appears to track inflammatory burden and oxidative protein damage at presentation.

Intra-redox associations. Beyond apoptosis-anchored correlations, the redox sub-system showed a strong coupling between reduced glutathione and protein carbonyls. GSH correlated positively with P-carb (μmol/L) (Spearman ρ = 0.73, p = 3.6×10^−5^, boot-strap 95% CI 0.47–0.87), and the association remained robust after age adjustment (partial Spearman ρ = 0.74, p = 3.4×10^−5^). A concordant relation was observed between P-carb (μmol/L) and P-carb (μmol/g protein) (ρ = 0.81, p < 10^−6^), serving as an internal consistency check. By contrast, GSH did not correlate meaningfully with TBARS or TAC (both p > 0.35). Taken together, these findings suggest that, at presentation, anti-oxidant buffering (GSH) co-varies with oxidative protein damage, consistent with a compensatory redox response rather than a lipid-peroxidation-driven pattern.

Exploratory derived indices. Redox ratios contrasting oxidative load against anti-oxidant buffer did not improve discrimination of radiologic severity: POBI = z(P-carb_g) − z(GSH) yielded AUC 0.48 (95% CI 0.19–0.77), LOBI = z(TBARS) − z(GSH) AUC 0.32 (0.10–0.57), and a focused variant of RII AUC 0.42 (0.17–0.68) (n = 25). Inflammation-independent protein oxidation (rank-residual P-carb_g | CRP) showed AUC 0.51 (0.22–0.79). By contrast, the redox network displayed a strong intra-redox coupling between GSH and protein carbonyls (ρ 0.73, bootstrap 95% CI 0.47–0.87; age-adjusted ρ 0.74), while TBARS did not correlate meaningfully with either GSH or P-carb (both p > 0.35). These patterns support a coupled antioxidant–protein-oxidation axis at presentation but do not translate into reliable severity discrimination on this sample.

To move beyond p-values, **Table 5** summarizes non-parametric effect sizes—Cliff’s delta (Δ) and the Hodges–Lehmann (HL) median shift (D minus A–C). Group sizes were identical across analytes (non-severe n=18; severe n=7), so N was omitted from the table for compactness. Effects were uniformly small (|Δ|≤0.27) and directionally aligned with the raw distributions; for illustration: AIM2 Δ=+0.27 (HL=−114.42 pg/mL; p=0.318), TBARS Δ=+0.16 (HL=−0.03 μmol/mL; p=0.565), CAT Δ=−0.16 (HL =+ 0.24 U/mL; p=0.574), IL-1β Δ=−0.16 (HL =+ 1.74 pg/mL; p=0.564), GSH Δ=−0.14 (HL =+ 0.09 μmol/L; p=0.628), CRP Δ=−0.10 (HL =+ 6.12 mg/L; p=0.745).

**Table 5:** Non-parametric effect sizes by severity (A–C vs D): Cliff’s delta (Δ) and Hodges–Lehmann median shift (D minus A–C), with BH–FDR q-values.

Marker	Median		Cliff’s Δ	HL shift (D–A–C)	P	Q
	Non-severe	Severe				
P-carb (μmol/L)	5.91	5.45	0.02	0.00	0.976	1.000
P-carb (μmol/g protein)	91.47	75.83	-0.02	1.27	0.976	1.000
TBARS (μmol/mL)	0.68	0.64	0.16	-0.03	0.565	1.000
TAC (μmol DPPH/L)	54.91	57.41	-0.03	1.48	0.929	1.000
CAT activity (U/mL)	4.69	4.97	-0.16	0.24	0.574	1.000
GSH (μmol/L)	1.30	1.46	-0.14	0.09	0.628	1.000
Caspase-3 (ng/mL)	7.37	7.07	0.00	-0.01	1.000	1.000
IL-1β (pg/mL)	10.11	12.82	-0.16	1.74	0.564	1.000
AIM2 (pg/mL)	857.15	772.86	0.27	-114.42	0.318	1.000
CRP (mg/L)	68.98	72.00	-0.10	6.12	0.745	1.000
SIRI (a.u.)	5.20	4.36	0.03	-0.08	0.929	1.000
AISI (a.u.)	1317.26	1179.77	-0.06	65.89	0.836	1.000

Δ interpreted as |Δ|≈0.11 small, ≈0.28 medium, ≈0.43 large; HL median shift reported as D mi-nus A–C. Multiple testing controlled using Benjamini–Hochberg FDR.

Categorical characteristics by radiologic severity (A–C vs D), including primary and secondary diagnoses, are summarized in **Table 6**. No categorical association reached statistical significance on two-sided Fisher’s exact tests despite imbalances and sparse cells: primary diagnosis (acute vs acute-on-chronic) p=1.000; secondary diagnosis (Balthazar D vs C) p=0.143; metabolic phenotype (pancreatitis-only vs pancreatitis+MetS) p=0.355; hypertension history p=0.407; MetS criteria p=0.355. Exploratory stratifications suggested higher inflammation-based indices in pancreatitis-only versus pancreatitis+MetS (AISI 1492.36 vs 391.14, p=0.066; MLR 0.51 vs 0.28, p=0.119; PLR 179.62 vs 87.50, p=0.149), whereas oxidative markers were broadly comparable. Consistent with the small effect-size profile and limited sample size, age-adjusted partial Spearman correlations between triglycerides and redox/inflammasome markers were small and non-significant (e.g., P-carb/g ρ_partial=−0.38, p=0.062; AIM2 ρ_partial=−0.37, p=0.067).

**Table 6:** Categorical characteristics by radiologic severity (A–C vs D).

Variable	Level	Non-severe n(%)	Severe n(%)
Primary diagnosis	Acute pancreatitis	15 (83.33%)	6 (85.71%)
	Acute-on-chronic pancreatitis	3 (16.67%)	1 (14.29%)
Secondary diagnosis (scoring)	Balthazar D	1 (5.56%)	3 (42.86%)
	Balthazar C	4 (22.22%)	0 (0.00%)
Metabolic phenotype	Pancreatitis only	10 (55.56%)	6 (85.71%)
	Pancreatitis + MetS	8 (44.44%)	1 (14.29%)
Hypertension history	Yes	9 (50.00%)	5 (71.43%)
	No	9 (50.00%)	2 (28.57%)
MetS criteria	No	10 (55.56%)	6 (85.71%)
	Yes	8 (44.44%)	1 (14.29%)

### 3.3 Predictive performance, calibration, and multivariable modeling

ROC performance for single markers is first summarized, followed by composite indices and combined multivariable models with internal optimism correction. Exploratory interaction terms, a data-driven oxidative score, and stratified sensitivity analyses are then reported. For interaction and Oxidative Burden models (**Tables 7**, **8**), we used the alternative split C/D vs B to mitigate class imbalance and qua-si-separation; all other analyses used D vs A–C.

**Table 7:** Oxidative Burden (z-Pcarb + z-TBARS − z-TAC − z-GSH) as predictor of radiologic se-verity (C/D vs B): odds ratios with 95% confidence intervals. Models adjusted as indicated; two-sided p values.

Model	Level	OR	95% CI	P
B1: OB + Age + Gender	Intercept	2.93	1.03–8.32	0.043
	Oxidative Burden	0.57	0.18–1.82	0.342
	Age (years)	2.44	0.81–7.34	0.114
	Gender	1.08	0.38–3.02	0.891
B2: OB + Age	Intercept	2.94	1.03–8.34	0.043
	Oxidative Burden	0.57	0.18–1.80	0.335
	Age (years)	2.41	0.81–7.14	0.112

Cross-validated AUCs for B1/B2 were reported in the analysis outputs (B1_CV/B2_CV) and are unstable (small sample).

#### 3.3.1 Single-marker ROC performance

Radiologic severity was defined as non-severe (A–C) versus severe (D). In this cohort (n = 25), single-marker discrimination at presentation was modest. The best tendencies were observed for GSH (AUC 0.57) and CRP (AUC 0.55), followed by the inflammatory composite AISI (AUC 0.53); for completeness, SIRI performed at AUC 0.48. Bootstrap 95% CIs were wide and all included 0.50, indicating absence of statistically secure separation: GSH 0.57 (0.24–0.85), CRP 0.55 (0.26–0.82), AISI 0.53 (0.30–0.75), SIRI 0.48 (0.24–0.75).

Across the broader oxidative/antioxidant/apoptotic/inflammatory panel, ROC traces clustered near the diagonal, and no key single marker exceeded ~0.60. Exploratory Youden thresholds were consistent with the modest signal and should be interpreted cautiously: GSH 1.46 μmol/L, CRP 101.00 mg/L, AISI 472.75 a.u. In contrast, several markers showed limited standalone performance around chance: TAC AUC 0.52, P-carb AUC 0.49, and caspase-3 AUC 0.50. Given the small sample and biological heterogeneity at first contact, these estimates primarily serve descriptive purposes rather than definitive cut-offs for clinical decision-making.

Convergent evidence from correlation analyses aligned with this small-effect profile. Spearman correlations between biomarkers and Balthazar inflammation points were small and non-significant, both unadjusted and age-adjusted: for GSH, ρ = 0.299, p = 0.147 (age-partial ρ = 0.224, p = 0.283); for AIM2, ρ = −0.227, p = 0.275 (age-partial ρ = −0.288, p = 0.163). Overall, the data indicate that—at presentation—neither systemic inflammatory tone nor redox status alone provides reliable discrimination of radiologic severity, and any thresholds derived from this dataset should be viewed as exploratory.

#### 3.3.2 Composite redox indices

To summarize multi-domain redox imbalance, we prespecified a composite RII computed on complete cases (n = 25) as: RII = z(P-carb) + z(TBARS) − z(TAC) − z(GSH) (z-scores computed in the study sample). As a single-score classifier of radiologic se-verity (coded D vs A–C for ROC analyses), RII showed limited discrimination with AUC 0.42 (bootstrap mean 0.424; 95% CI 0.17–0.69). The Youden cut-off was −2.63 a.u., yielding sensitivity 1.00 and specificity 0.11 (LR + 1.13, LR− 0.00). Taken together, these characteristics indicate an extremely liberal threshold—nearly all severe cases are flagged, but at the expense of many false positives. Because the confidence interval spans 0.50 and specificity is very low, the evidence supports a near-chance signal for RII in this dataset, consistent with the small-effect pattern seen for its individual com-ponents.

For regression-based analyses we further evaluated a prespecified composite un-der the label “Oxidative Burden” (same z-standardized construction). To mitigate class imbalance and quasi-separation in multivariable settings, these models used the alternative C/D vs B split (see **Table 7** for odds ratios with 95% CIs and p-values). In line with the ROC findings, Oxidative Burden did not yield robust, clinically actionable separation; rather, the estimates were imprecise and highly sensitive to sample sparsi-ty. Overall, the RII/Oxidative Burden results suggest that, at presentation, integrated redox dysregulation captures some trend toward more severe disease but falls short of reliable discrimination in this cohort.

#### 3.3.3 Combined models and calibration

Logistic regression models were fit with predictors z-scored and radiologic sever-ity coded D vs A–C (n = 25). A parsimonious four-marker specification (caspase-3 + CRP + SIRI + AISI) yielded an apparent AUC of 0.59, which declined to AUC 0.39 after 0.632 bootstrap optimism correction.

Inclusion of the redox composite RII did not enhance performance (apparent 0.59, corrected 0.37).A leaner three-marker model (CRP + GSH + AISI) showed a similar profile (apparent AUC0.56,.632-corrected 0.39). Across specifications, no individual predictor reached statistical significance (all p ≥ 0.05, two-sided) (**Table 9**).

The convergence of validated AUCs toward ~0.39–0.40 across model variants indicates overfitting at this sample size, plausibly related to low events-per-variable and correlated predictors drawn from overlapping inflammatory and redox pathways. The discrepancy between apparent and optimism-corrected performance suggests that in-sample separation is not reproducible under resampling. Taken together with single-marker results, these findings indicate that, at presentation, the available apoptotic, inflammatory, and redox markers convey limited and imprecise discriminatory in-formation for radiologic severity in this cohort. Overall, while mechanistically coherent associations were observed, internal validation indicates that admission-time biomarker models do not yield a stable prediction rule for disease severity in this dataset. We therefore refrain from proposing clinical cut-offs or a scoring tool and present these results as hypothesis-generating for time-resolved panel validation. Larger cohorts, leaner prespecified signatures, and external validation would be required to stabilize estimates and to clarify whether modest effects observed here can be translated into clinically useful prediction. Consistent with the small sample size and low events-per-variable, optimism-corrected AUC values dropped to ~0.39, indicating that the models lack stable predictive performance.

#### 3.3.4 Interaction terms and data-driven score

Interaction terms between age and key antioxidant markers did not reach statistical significance. In models fit on the C/D vs B split, the Age×GSH interaction (Model I1) was non-significant (p = 0.916), as was Age×TAC (Model I2; p = 0.699). Main effects of age were positive and reached nominal significance in both models (I1 p = 0.047; I2 p = 0.044), albeit with wide confidence intervals, indicating imprecision. Discrimination was poor to modest: Model I1 had an apparent AUC of 0.44, while cross-validated AUCs for both interaction models were low and unstable, consistent with small sample size and class imbalance (**Tables 8A**, **B**).

**Table 8A:** Logistic regression with interaction terms (Balthazar C/D vs B) — Model I1 (AgeClass55 × GSH).

Predictor	OR	95% CI	P
Intercept	7.69	0.49–120.55	0.146
Age (years)	51.27	1.04–2520.14	0.047
AgeClass55	0.05	—	0.653
GSH (μmol/L)	13.75	—	0.385
AgeClass55 × GSH	0.42	—	0.916

**Table 8B:** Logistic regression with interaction terms — Model I2 (AgeClass55 × TAC).

Predictor	BOR	95% CI	P
Intercept	8.20	0.88–66.85	0.079
Age (years)	25.67	0.63–1043.99	0.044
AgeClass55	0.23	—	0.758
TAC (μmol DPPH/L)	8.51	—	0.236
AgeClass55 × TAC	3.81	—	0.699

**Table 9:** Binary logistic regression for severe pancreatitis (Severe ~ CRP + SIRI).

Predictor	OR	95% CI	P
CRP (mg/L)	0.999	0.988–1.010	0.820
SIRI	1.001	0.747–1.341	0.996

Model performance: AUC 0.44; n = 25.

A principal-component score summarizing oxidative markers indicated that PC1 captured 44.8% ofvariance, driven primarily by protein carbonyls, with minor contributions from GSH and CAT, and negligible TAC loading. In an age-adjusted logistic model, the PC1-derived oxidative score showed a positive but non-significant association with severity (OR 2.58, 95% CI 0.63–10.64; p = 0.190), whereas age remained significant (OR 3.49, 95% CI 1.01–12.07; p = 0.048). Cross-validated discrimination was modest and unstable (AUC 0.72 ± 0.30), underscoring limited reproducibility at this sample size (**Table 10**).

**Table 10:** Logistic regression with data-driven oxidative score (PC1).

Predictor	β (SE)	OR	95% CI (lower – upper)	P
Intercept	1.23 (0.60)	3.42	1.06–11.05	0.040
Age (years)	1.25 (0.63)	3.49	1.01–12.07	0.048
PC1_oxidative	0.95 (0.72)	2.58	0.63–10.64	0.190

#### 3.3.5 Stratified sensitivity analyses (gender and age)

Gender–stratified comparisons did not identify statistically significant differences across clinical or biochemical domains (all p>0.20). Normalized protein carbonyls (μmol/g protein) tended to be higher in females (median 100.17 [95.72–173.30] vs 74.51 [65.66–141.28]; U = 92.0; p=0.085), while distributions of age class, pancreatitis type, Balthazar grades, BMI categories, metabolic phenotype, hypertension, and diabetes were otherwise comparable (all p≥0.29).

By age class (≤55 vs ≥55 years), pancreatitis type and Balthazar grades showed no statistically significant variation (both p≥0.27), and BMI categories were balanced (p=1.000). A significant association was observed between age class and metabolic phenotype (pancreatitis only vs pancreatitis+MetS), with older patients more frequently classified as pancreatitis only and younger patients as pancreatitis+MetS (Fisher’s exact p=0.041). Hypertension and diabetes distributions were comparable across age groups (all p>0.12).

## 4 Discussion

Although caspase-3 correlated with CRP, GSH, and P-carb, this cross-sectional signal did not translate into an effective admission prediction tool. This gap is expected in small heterogeneous cohorts, where single time-point measurements, correlated predictors, and low events-per-variable inflate optimism and reduce reproducibility after internal validation. This cohort delineates a coherent redox–inflammation–apoptosis profile at presentation in acute pancreatitis (AP). Moderate, age-robust correlations linked caspase-3 with CRP, GSH, and protein carbonyls (P-carb), and the intra-redox association of GSH–P-carb was strong. In contrast, individual markers (including CRP, GSH, AISI, SIRI, P-carb, TBARS, TAC) showed modest discrimination of radiological severity (AUC ≈ 0.48–0.57; all CIs include 0.50), as did RII and derived “load-vs-buffer” indices (POBI/LOBI). Combined models overfitted after.632 correction, and predefined inter-actions and PC1-oxidative performed inconsistently. Taken together, this suggests that, at present, the clinical utility of these biomarkers is more for biological phenotyping than for severity stratification.

The absence of statistically significant differences between severity groups and the poor discriminatory performance of both single markers and composite indices should be interpreted in the context of the study design. First, blood sampling was performed at a single early time point (admission). Oxidative stress in acute pancreatitis is highly dynamic; markers of lipid peroxidation and antioxidant capacity can change markedly within the first 48–72 h. A cross-sectional design therefore cannot capture the kinetic evolution of redox imbalance. Second, the cohort was heterogeneous with respect to etiology, presence of metabolic syndrome, and acute versus acute-on-chronic presentations, all of which may influence baseline redox status. Third, several potential confounders (smoking, alcohol consumption, pre-admission medications) were incompletely documented and could not be fully controlled.

These factors collectively explain why the tested biomarkers, although mechanistically relevant, failed to discriminate radiologic severity at presentation in this pilot cohort. The observed biological signals (strong GSH–P-carb coupling and caspase-3 correlations with CRP and protein carbonyls) nevertheless remain coherent with current understanding of AP pathogenesis and warrant further investigation in longitudinal protocols.

The robust GSH-P-carb correlation indicates a profile dominated by protein oxidation upon presentation. Mechanistically, protein carbonyls reflect irreversible protein alterations by ROS and are recognized as canonical markers of oxidative stress; they can occur early during AP and have been reported elevated in severe forms, although their early prognostic value remains inconclusive (1). In mirror image, GSH is the main intracellular thiol buffer; its depletion is linked to activation of pro-oxidative pathways and inflammation. Clinical and experimental data show that GSH status can decrease in pancreatitis, but the magnitude and dynamics depend on the temporal and clinical context (2).

The observation that TBARS aligns poorly with GSH/P-carb in this setting is con-sistent with the literature describing the variability of lipid peroxidation in human PA and its sensitivity to time window of harvest, compartment (serum vs. tissue/ascites), and comorbidities. Some studies report increases in MDA/TBARS, but the relationship with clinical severity is uneven (3). Overall, our pattern-antioxidant-predominant protein oxidation, with lipid peroxidation less informative at presentation-is consistent with recent reviews that place oxidative stress at the center of PA pathogenesis but emphasize the kinetic complexity of redox markers (4).

The apoptosis-inflammation-redox axis is supported by caspase-3 correlations with CRP/GSH/P-carb. Mechanistically, caspase-3 integrates intrinsic/extrinsic signalling of apoptosis, is activated in AP, and may interact with inflammatory pathways (inflammasome/NLRP) (5). Thus, the alignment observed in our data is biologically plausible: an altered redox status (increased protein oxidation and GSH buffering) may go in parallel with apoptotic activation and amplification of systemic inflammation.

The results are in line with the literature supporting the involvement of oxidative stress in PA in humans, including increases in P-carb in severe forms and early onset of protein oxidation (6). At the same time, the current data do not support the use of redox markers for robust severity screening at presentation-a conclusion consistent with reviews of biomarkers of severity and controversial evidence on CRP as an early predictor (7). Moreover, recent observations emphasize the role of the NLRP3 inflammasome and its interface with apoptosis and redox signaling, supporting our mechanistic interpretation (8). NLRP3 inflammasome are frequently studied as ROS and cellular danger sensor that promote early inflammation by increased IL1β and IL18 levels. Among inflammasomes, AIM2 inflammasome is less studied in clinical trials. Similar with NLRP3 inflammasome, AIM2 act as sensor of dsDNA released by dam-aged cells initiating IL1β and IL18 mediated inflammation and pyroptotic cell death by caspase 1 activation. Oxidative stress unbalance can direct or indirect contribute to in-flammasomes activation by altering host cells and release mitochondrial or cellular dsDNA that act as DAMPs. What we know up to date from animal model and human studies of pancreatitis is the fact that AIM2 suppression decrease pancreatic necrosis, serum amylase and IL1β production (30). Our study did not show a statistic significant correlation of AIM2 inflammasome with inflammation or diseases severity. An explanation for this can be linked with small sample size or single time point analysis, but inflammasome involvement in AP remain challenging.

Regarding antioxidants, clinical trials remain mixed: meta-analyses and systematic syntheses show no consistent benefit on clinical severity or progression in PA, although there may be specific time/dose specific signals or particular contexts (e.g. post-ERCP) (9). This landscape reinforces the idea that parsimonious panels and timing of harvests may be more relevant than a singular marker measured at a single presentation.

From a translational perspective, the redox markers of this study offer phenotyping value: (i) an antioxidant-protein oxidation profile (GSH-P-carb) that can serve as a mechanistic signature; (ii) an alignment of apoptosis (caspase-3) with inflammation and redox; (iii) borderline utility for sorting radiologic severity at presentation, in agreement with the clinical literature on CRP and other general biomarkers. In practice, redox may be more suitable for biological profiling and monitoring (e.g., trajectories in the first 48–72 h), or as an addition to clinical/imaging scores, than as a single screening test.

For future clinical integration, we recommend serial sampling at admission (0 h) and at 24, 48, and 72 h. Multi-time-point integration should prioritize dynamic features rather than single thresholds: (i) absolute change (Δ0–24 h, Δ24–48 h), (ii) slope over 0–72 h, (iii) peak value within 72 h, and (iv) area under the biomarker trajectory. These features can be combined with clinical/imaging scores in mixed-effects or time-updated logistic models to support dynamic risk prediction.

We adjusted primary association analyses for age (and sex in predictive models), yet other confounders such as etiology, comorbidities, smoking/alcohol exposure, and medication history may influence redox and inflammatory markers. Where available, we addressed this through parsimonious sensitivity analyses (etiology/comorbidity burden), but residual confounding remains possible.

### 4.1 Limitations of the study

The present study has several limitations that should be considered when interpreting the results. First, the small sample size (n = 25), with only 7 patients in the severe radiologic group (Balthazar D), substantially limits statistical power and the reliability of multivariable analyses and ROC discrimination estimates. *Post-hoc* power calculations based on the observed small-to-modest effect sizes (Cliff’s Δ ≤ 0.27 for most markers) indicate that approximately 80–120 total patients would be required to detect group differences in key biomarkers such as GSH or CRP with 80% power at α = 0.05. This confirms the pilot nature of the work and underscores the need for larger validation cohorts.

Second, blood sampling was performed at a single time point (emergency department admission, prior to specific therapy). Oxidative stress and inflammatory responses in acute pancreatitis are highly dynamic processes that evolve rapidly within the first 48–72 hours. A cross-sectional design therefore cannot capture the kinetic changes or peak values of redox and inflammatory markers. In addition, the exact time from symptom onset to blood sampling was not systematically recorded in all cases, representing a further limitation.

Third, several potential confounding factors, including smoking status, alcohol consumption, pre-admission medications (e.g., statins, antioxidants, or corticosteroids), and detailed etiology documentation, were incompletely captured in the medical records and could not be fully adjusted for. Although we applied age-adjusted partial correlations and parsimonious sensitivity analyses where possible, residual confounding cannot be excluded.

Finally, the combined logistic regression models showed substantial overfitting, with apparent AUC values of 0.56–0.59 dropping to approximately 0.39 after.632 bootstrap correction. We addressed optimism using bootstrap resampling and complete-case analysis, and we controlled multiplicity with the Benjamini–Hochberg FDR procedure for prespecified correlation families. Nevertheless, these statistical approaches, while appropriate for small pilot datasets, are not a substitute for external validation in larger, prospective studies.

Despite these limitations, the study provides useful preliminary insights into the antioxidant–protein oxidation axis and its relationship with apoptosis and systemic inflammation at disease presentation. The findings should be regarded as hypothesis-generating and will inform the design of our planned larger, longitudinal protocol with serial sampling.

## 5 Conclusions

This small observational pilot study demonstrates a biologically coherent redox–inflammation–apoptosis coupling at presentation in acute pancreatitis. We observed a strong intra-redox association between reduced glutathione (GSH) and protein carbonyls, together with moderate, age-robust correlations between caspase-3 and both C-reactive protein (CRP) and protein carbonyls.

However, neither individual oxidative stress, antioxidant, inflammatory, nor apoptotic markers, nor the prespecified Redox Imbalance Index, reliably discriminated radiologic severity (Balthazar D vs A–C) at admission. Single-marker discrimination was modest (AUCs 0.42–0.57, with all 95% confidence intervals including 0.50), and multivariable logistic models showed substantial overfitting after.632 bootstrap correction (corrected AUC ≈ 0.39).

These findings indicate that the tested biomarkers do not support the development of a stand-alone admission triage tool in this cohort. Their main value lies in providing mechanistic insights into the antioxidant–protein oxidation axis and its relationship with apoptosis and systemic inflammation. The results motivate larger, adequately powered, multicenter prospective studies that incorporate serial blood sampling (e.g., at 0 h, 24 h, 48 h, and 72 h) to characterize the temporal dynamics of these markers and to evaluate parsimonious, pre-specified biomarker panels integrated with clinical and imaging-based scores.

## Data Availability

The original contributions presented in the study are included in the article/supplementary material. Further inquiries can be directed to the corresponding authors.
